# Long-term outcomes after endoscopic retrograde pancreatic drainage for symptomatic pancreaticojejunal anastomotic stenosis

**DOI:** 10.1038/s41598-021-84024-z

**Published:** 2021-02-24

**Authors:** Akihiko Kida, Yukihiro Shirota, Taro Kawane, Hitoshi Omura, Tatsuo Kumai, Masaaki Yano, Fumitaka Arihara, Yuji Hodo, Koichiro Matsuda, Kohei Ogawa, Mitsuru Matsuda, Akito Sakai, Mitsuhiro Terada, Tokio Wakabayashi

**Affiliations:** 1 Department of Gastroenterology, JA Toyama Kouseiren Takaoka Hospital, 5-10 Eirakuchou, Takaoka, Toyama, 933-0843 Japan; 2 Department of Gastroenterology, Saiseikai Kanazawa Hospital, 13-6 Akatsuchimachi, Kanazawa, Ishikawa 920-0353 Japan; 3 Department of Internal Medicine, Toyama Prefectural Central Hospital, 2-2-78 Nishinagae, Toyama, 930-8550 Japan

**Keywords:** Anatomy, Diseases, Gastroenterology, Medical research

## Abstract

There is limited evidence supporting the usefulness of endoscopic retrograde pancreatic drainage (ERPD) for symptomatic pancreaticojejunal anastomotic stenosis (sPJS). We examined the usefulness of ERPD for sPJS. We conducted a retrospective analysis of 10 benign sPJS patients. A forward-viewing endoscope was used in all sessions. Following items were evaluated: technical success, adverse events, and clinical outcome of ERPD. The technical success rate was 100% (10/10) in initial ERPD; 9 patients had a pancreatic stent (no-internal-flap: n = 4, internal-flap: n = 5). The median follow-up was 920 days. Four patients developed recurrence. Among them, 3 had a stent with no-internal-flap in initial ERPD, the stent migrated in 3 at recurrence, and a stent was not placed in 1 patient in initial ERPD. Four follow-up interventions were performed. No recurrence was observed in 6 patients. None of the stents migrated (no-internal-flap: n = 1, internal-flap: n = 5) and no stents were replaced due to stent failure. Stenting with no-internal-flap was associated with recurrence (*p* = 0.042). Mild adverse events developed in 14.3% (2/14). In conclusions, ERPD was performed safely with high technical success. Recurrence was common after stenting with no-internal-flap. Long-term stenting did not result in stent failure.

Clinical trial register and their clinical registration number: Nos. 58-115 and R2-9.

## Introduction

Pancreaticojejunal anastomotic stenosis (PJS) is an important late complication associated with pancreaticoduodenectomy (PD), with a reported incidence rate of 1.4–11.4%^1^. The most common symptoms of PJS were abdominal pain and recurrent acute pancreatitis, followed by impaired pancreatic function^1^. Interventions for symptomatic PJS (sPJS) include surgery and endoscopic procedures. Surgery is performed as a revision to the initial PD and is thus considered highly invasive. Therefore, endoscopic interventions are often preferred as the initial treatment strategy for sPJS. Endoscopic retrograde pancreatic drainage (ERPD) and endoscopic ultrasound-guided pancreatic duct drainage (EUS-PD), including the rendezvous technique using EUS-guided puncture, are commonly reported as effective endoscopic interventions for sPJS. Recently, EUS-PD is the preferred treatment for sPJS^2^. There are several reasons for selecting EUS-PD over ERPD. For example, a multicenter international collaborative study demonstrated that EUS-PD is an effective treatment strategy with a surgical success rate of 89% (71/80) and clinical efficacy of 81% (65/80)^3^. On the other hand, ERPD requires identification and cannulation of the anastomotic site, which is technically challenging. As a result, the reported success rate of ERPD is below 30%^4–7^. However, EUS-PD is associated with a relatively high rate of adverse events (AE) of 20% and these AE are known to be moderate to severe^3^. Considering the high success rate and risk of severe AE demonstrated in the retrospective study, it is recommended that only experts with technical competency perform EUS-PD^3^. On the other hand, we previously reported in a relatively small patient population that ERPD is a safe and effective treatment for sPJS^8^.

In the present study, we report 10 consecutive cases of sPJS. The anastomotic site was identified endoscopically and ERPD was performed successfully in all cases. Our study suggests that, in contrast to previous reports, ERPD is technically feasible as an endoscopic intervention for sPJS. Furthermore, we were able to perform long-term follow-up of patients after ERPD to examine the incidence of sPJS recurrence. Thus, we retrospectively examined the efficacy and safety of ERPD for sPJS in 10 patients.

## Results

### Patient characteristics

A total of 10 patients who were diagnosed with sPJS underwent ERPD. The patient characteristics are summarized in Table 1. The study subjects had a median age of 64.5 (45–83), and consisted of 6 male and 4 female patients. Surgical procedures and reconstruction methods employed included pylorus-preserving pancreaticoduodenectomy (PpPD) with modified-Child’s reconstruction (n = 8), PpPD with Cattell’s reconstruction (n = 1), and subtotal stomach-preserving PD (SSpPD) with modified-Child’s reconstruction (n = 1). The cause of sPJS was benign stenosis in all 10 patients.

**Table 1:** Characteristics of patients.

No. of patients	10
Age, years	64.5 (45–83)
Sex, M/F	6/4
**Surgical procedure and reconstruction method employed**	
PpPD with modified-child’s/Cattell’s reconstruction	8/1
SSpPD with modified-child’s reconstruction	1
**Etiology requiring surgery**	
Pancreatic cancer	3
Intraductal papillary mucinous neoplasm	4
Biliary tract cancer	2
Papillary carcinoma of the duodenum	1
**Cause of PJS**	
Benign stenosis	10
Clinical symptom of sPJS	
Recurrent acute pancreatitis	8
Glucose intolerance and exocrine pancreatic insufficiency	2

*M* male, *F* female, *PpPD* pylous-preserving pancreaticoduodenectomy, *SSpPD* subtotal stomach-preserving pancreaticoduodenectomy, *PJS* pancreaticojejunal anastomotic stenosis, *sPJS* symptomatic pancreaticojejunal anastomotic stenosis.

### Reaching the anastomotic site with the endoscope and endoscopic identification of the anastomotic site

**Figure 1:**
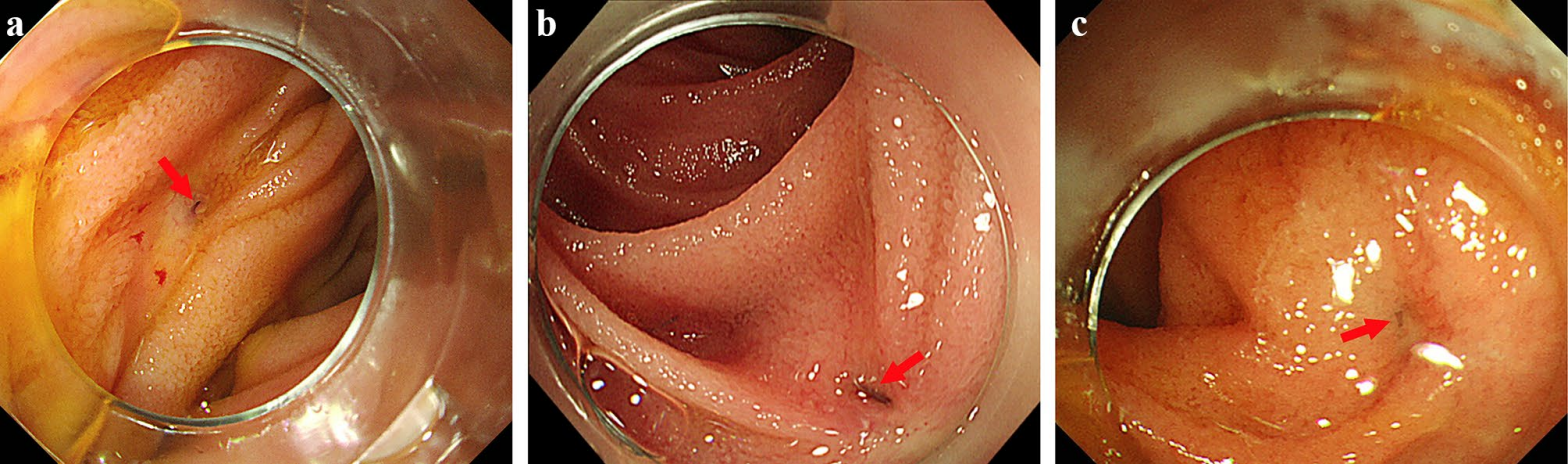
Endoscopic findings at the anastomotic site. (**a**) The anastomotic site with pinhole-like opening was located frontward to the endoscopic view. The red arrow shows the anastomotic site. (**b**) The anastomotic site with slit-like opening was located tangent to the endoscopic view. The red arrow shows the anastomotic site. (**c**) The anastomotic site with scarred, membranous stenosis was located tangent to the endoscopic view. The red arrow shows the anastomotic site.

The anastomotic site was accessible and identified in all 10 patients (14 sessions) by forward-viewing endoscopes. A balloon-assisted endoscope was used in 3 of all sessions. A tip attachment was applied to the endoscope in all sessions to facilitate identification of the anastomotic sites. The anastomotic site position and characteristics are summarized in Table 2. In 9 patients who underwent PpPD and SSpPD with modified-Child’s reconstruction, the anastomotic sites were located tangent to the endoscopic view. In the remaining 1 patient who underwent PpPD with Cattell’s reconstruction, the anastomotic site was located frontward to the endoscopic view. Anastomotic sites were characterized by pinhole-like opening (n = 1), slit-like opening (n = 3), and scarred, membranous stenosis (n = 6). The representative position and characteristics of anastomotic site are shown in Fig. 1. In 1 patient whose anastomotic site was characterized by scarred, membranous stenosis, the site was confirmed by imaging the remnant main pancreatic duct by EUS using a small-diameter probe with a 2050-mm working length (UM-G20-29R; Olympus Medical Systems, Tokyo, Japan). A forward-viewing endoscope with a 1330-mm working length (PCF-PQ260I; Olympus Medical Systems) was used in this patient. In the other 3 patients whose anastomotic site was characterized by scarred, membranous stenosis, the sites were confirmed by pancreatography of the remnant main pancreatic duct using an injection needle (23-G Top endoscopic injection needle; TOP, Tokyo, Japan). The anastomotic site was carefully punctured using an injection needle predicting the path of the remnant main pancreatic duct under fluoroscopy. After puncturing the anastomotic site, contrast medium was slowly injected into the remnant main pancreatic duct, and 3 or 4 punctures were required for pancreatography.

**Table 2:** Anastomotic site of sPJS.

Successful identification of anastomotic site	10/10
**Anastomotic site position (Tangent/frontward to the endoscopic view)**	
PpPD and SSpPD with modified-child’s reconstruction	9/0
PpPD with Cattell’s reconstruction	0/1
**Anastomotic site characteristics**	
Pinhole-like opening/slit-like opening/scarred, membranous stenosis	1/3/6
**Procedural modification to identify the anastomotic site**	
Use of a tip attachment	10
Imaging of the remnant pancreatic duct by endoscopic ultrasound with a small-diameter probe	1
Pancreatography of the remnant pancreatic duct by an injection needle	3

*sPJS* symptomatic pancreaticojejunal anastomotic stenosis, *PpPD* pylous-preserving pancreaticoduodenectomy, *SSpPD* subtotal stomach-preserving pancreaticoduodenectomy.

### Initial ERPD for sPJS

Endoscopic retrograde pancreatography (ERP) was performed and a guidewire was placed successfully in all 10 patients. The methods of ERP and guidewire placement for each anastomotic site are summarized in Table 3. In 4 patients whose anastomotic site was characterized by a pinhole- or slit-like opening, ERP and guidewire placement were performed with cannulation alone or facilitated by the use of wire-guided technique (WGT). In 3 of 6 patients whose anastomotic site was characterized by scarred, membranous stenosis, ERP and guidewire placement required highly technical procedures such as incision of the anastomotic site with a needle knife (KD-1L-1; Olympus Medical Systems).

**Table 3:** ERPD.

Successful ERP and placement of a guidewire	10/10
**ERP and placement of a guidewire by anastomotic site characteristics**	
Anastomotic site with pinhole-like opening	1
Concurrent use of WGT	1
Anastomotic site with slit-like opening	3
Cannulation alone/Concurrent use of WGT	2/1
Anastomotic site with scarred, membranous stenosis	6
Cannulation alone/concurrent use of WGT/concurrent use of WGT + incision of the anastomotic site with a needle knife/concurrent use of injection needle + WGT/concurrent use of injection needle + WGT + incision of the anastomotic site with a needle knife	1/1/1/1/2
Successful ERPD	10/10
Dilation of the anastomotic site	10
Dilation with a contrast cannula/balloon/dilator	2/7/1
Balloon dilation of the anastomotic site	7
The median ratio of diameters of balloon to that of remnant main pancreatic duct	0.86 (0.47–1.60)
Waist disappearance: yes/no	5/2
Pancreatic calculus	2
Removed: yes/no	2/0
Pancreatic stent placement	9
Type (with no-internal-flap/with internal-flap)	4/5
Size (4/5/7 Fr)	1/6/2

*ERPD* endoscopic retrograde pancreatic drainage, *ERP* endoscopic retrograde pancreatography, *WGT* wire-guided technique.

Following placement of a guidewire, anastomotic sites were dilated in all 10 patients. The details of ERPD are summarized in Table 3. Anastomotic sites were dilated with a balloon (n = 7), a dilator (n = 1), and a contrast cannula (n = 2). A pancreatic stent was placed in 9 patients (with no-internal-flap: n = 4, with internal-flap: n = 5). Pancreatic calculi were removed from 2 patients.

### Recurrence of sPJS after ERPD

The median follow-up after initial ERPD was 920 days (137–2223 days). Of the 10 patients, 4 developed recurrent sPJS in a median of 540 days (33–1865 days). The characteristics of the 4 recurrent sPJS patients are summarized in Table 4. Pancreatic stents with no-internal-flap were used in 3 of 4 recurrent sPJS patients in initial ERPD, and the stent migrated in all 3 patients at recurrence. A pancreatic stent was not placed in the remaining 1 patient in initial ERPD. A total of 4 follow-up interventions were performed in 3 of 4 patients with recurrent sPJS. At recurrence, significant scarring was observed at 3 anastomotic site. Two patients underwent follow-up ERPD including incision of the anastomotic site with a needle knife, and 1 patient required 2 follow-up interventions including EUS-guided retrograde pancreatic drainage through the anastomotic site using a forward-viewing echoendoscope (TGF-UC260J; Olympus Medical Systems)^9^. The remaining 1 patient declined follow-up interventions.

**Table 4:** Recurrent sPJS after ERPD.

Median follow-up after initial ERPD (n = 10, days)	920 (137–2223)
sPJS recurrence: yes/no	4/6
Median follow-up to sPJS recurrence (n = 4, days)	540 (33–1865)
**Characteristics of sPJS recurrence (n = 4)**	
Anastomotic site in initial ERPD: pinhole/slit/scarred, membranous stenosis	0/2/2
Balloon dilation in initial ERPD: yes/no	4/0
Waist disappearance by balloon dilation in initial ERPD: yes/no	2/2
Pancreatic calculus in initial ERPD: yes/no	1/3
Pancreatic stent placement in initial ERPD: yes/no	3/1
Type of pancreatic stent: with no-internal-flap/with internal-flap	3/0
Size of pancreatic stent: 4/5/7 Fr	0/2/1
Pancreatic stent migration at recurrence: yes/no	3/0

*sPJS* symptomatic pancreaticojejunal anastomotic stenosis, *ERPD* endoscopic retrograde pancreatic drainage.

Six patients did not have recurrent sPJS. The median follow-up in these patients was 292 days (137–1000 days). None of the pancreatic stents placed in these patients migrated and no pancreatic stents were replaced during the follow-up period. Among them, 5 patients had pancreatic stents with an internal-flap and 1 had a pancreatic stent with no-internal-flap.

On univariate analysis, the use of a pancreatic stent with no-internal-flap was identified as a risk factor for sPJS recurrence (*p* = 0.042) (Table 5).

**Table 5:** Factors associated with sPJS recurrence.

Factors associated with sPJS recurrence	*p*
Age (≥ 64.5 years vs < 64.5 years)	0.060
Sex (male vs female)	0.752
Characteristics of the anastomotic site (scarred, membranous stenosis vs pinhole + slit)	0.998
Pancreatic stent placement (yes vs no)	0.142
Type of pancreatic stent (with no-internal-flap vs with internal-flap)	0.042
Size of pancreatic stent (4 Fr + 5 Fr vs 7 Fr)	0.317
ERPD-associated AE (yes vs no)	0.808
Pancreatic stent migration (yes vs no)	0.062

*sPJS* symptomatic pancreaticojejunal anastomotic stenosis, *ERPD* endoscopic retrograde pancreatic drainage, *AE* adverse events.

### AE after endoscopic interventions

AE developed in 14.3% of all sessions (2/14). These AE were mild post endoscopic retrograde pancreatography pancreatitis (PEP) (n = 1) and anastomotic leak due to balloon dilation (n = 1). No AE caused by ERP using the injection needle were observed. The patient with mild PEP did not have a pancreatic stent placed after initial ERPD. In the second patient, the anastomotic leak was identified after the patient presented with abdominal pain after initial ERPD. Both of these AE were treated conservatively.

## Discussion

ERPD is generally considered challenging to perform in patients with sPJS^10^. Our study suggested that the following are essential in improving the technical success of ERPD: (1) Reaching the anastomotic site, and (2) identification of the anastomotic site and ERP of the remnant main pancreatic duct, followed by the placement of a guidewire.

Reaching the anastomotic site in sPJS is considered technically challenging because the anastomotic site is located deep in the afferent limb, or the afferent limb has a long length or marked intestinal adhesion. In our study, we were able to reach the anastomotic site using a forward-viewing endoscope including a balloon-assisted endoscope in all sessions. Recently, device-assisted endoscopes, such as balloon-assisted endoscopes and spiral endoscopes, have enabled endoscopic access deep into the afferent limb^11^. In addition, in a study of 38 cases of pancreatic diseases with surgically altered anatomy (PJS were 18 cases), the anastomotic site was reached in 37 using double-balloon endoscope^12^. Therefore, insertion into the anastomotic site by a forward viewing endoscope is considered useful.

However, identification of the anastomotic site and pancreatic cannulation is difficult because the anastomotic site locates the position which is difficult in approaching and it is originally small and close to obstruction such as scarred, membranous stenosis. It was also reported that the success rate of pancreatic cannulation in patients with surgically altered anatomy is 72%^12^. In our study, 9 of 10 anastomotic sites were located tangent to the endoscopic view, and 6 of 10 exhibited scarred, membranous stenosis. Therefore, it is important to understand that the position and characteristics of anastomotic site may differ due to surgical procedures and reconstruction methods employed. Furthermore, it may be beneficial to identify the remnant main pancreatic duct on either EUS or pancreatography using an injection needle to confirm the anastomotic site, and highly technical procedures, such as incision of the anastomotic site with a needle knife and EUS-guided retrograde pancreatic drainage, may be beneficial in pancreatic cannulation^9,13–15^.

We performed long-term follow-up of patients who underwent ERPD, and found that sPJS recurred in patients who had no pancreatic stents placed in initial ERPD and in those whose pancreatic stents migrated. Stent migration was specific to pancreatic stents with no-internal-flap in our patients and the use of pancreatic stents with no-internal-flap was associated with sPJS recurrence. If pancreatic stents with no-internal-flap are placed, stent migration can be expected. We initially thought that if the anastomotic site was successfully dilated, restenosis of the anastomotic site would not occur and sPJS recurrence would be low. Therefore, pancreatic stents with no-internal-flap were placed to prevent PEP. Although only a small case series was examined in our study, sPJS recurrence was more likely to occur after pancreatic stent placement with no-internal-flap. In another study of long-term outcomes after ERPD for sPJS, 6 patients with improved sPJS after pancreatic stenting had pancreatic stents removed and 3 had sPJS recurrence within 1 year^12^. In addition, in a similar study of 6 sPJS patients, 2 had sPJS recurrence within 2 years after stent removal^16^. Based on the above studies, pancreatic stent placement with an internal-flap may be useful to prevent sPJS recurrence.

In our study, none of the patients without sPJS recurrence required replacement of their stents within the follow-up period of approximately 10 months. However, none of the patients developed sPJS recurrence due to occlusion or failure of their stents. Previous studies revealed that long-term placement of pancreatic stents for patients with chronic pancreatitis can result in stent occlusion and subsequent pancreatitis within at least 6 months after pancreatic stenting^17–19^. Thus, it is common to replace pancreatic stents every 3 months^20^. The clinical pathogenesis of sPJS that developed as a result of postoperative benign stenosis of the anastomotic site is different from that of chronic pancreatitis characterized by the reduced output of pancreatic juice or by the presence of pancreatic calculi and mucous plug that developed as a result of the increased viscosity of pancreatic juice. These differences may underlie the relatively low risk of stent occlusion or failure due to long-term placement of a pancreatic stent in patients with sPJS. Thus, patients with sPJS may not require regular pancreatic stent replacement every 3 months, which is otherwise recommended for patients with chronic pancreatitis.

Several limitations of this study must be acknowledged. First, this study was a retrospective study. Second, as this study consisted of data from only two institutions, selection bias may have affected the patient characteristics and treatment choice. Third, these data were retrospectively extracted from medical records and undescribed data were inevitable limitations.

In conclusion, we demonstrated that ERPD can be performed safely with a high technical success rate for patients with sPJS based on appropriate identification of anastomotic sites and procedural modifications. Recurrence of sPJS was more common in patients who had a pancreatic stent with no-internal-flap, which was associated with a high risk of migration. Furthermore, long-term placement of pancreatic stents did not result in occlusion or failure of stents during the follow-up period.

## Materials and methods

### Study design

This was a retrospective analysis of patients with sPJS who underwent ERPD between April 2014 and May 2020. We identified consecutive patients with sPJS through the ERCP database, the EUS database, the registration system for national insurance reimbursement claims and the discharge summaries at Toyama Prefectural Central Hospital and Saiseikai Kanazawa Hospital. The primary endpoint was technical success of ERPD. The secondary endpoints were clinical outcome and procedure-related AE of ERPD.

Written informed consent was received from each patient before endoscopic treatment. This study was approved by the ethics committees of Toyama Prefectural Central Hospital (No. 58-115) and Saiseikai Kanazawa Hospital (No. R2-9), and was conducted in accordance with the ethical standards described in the latest revision of the Declaration of Helsinki. Informed consent for patient participation was received in the form of an opt-out in-hospital notice.

### Definition of sPJS

The patients with sPJS were defined as those who had clinical symptoms such as pancreatitis, exocrine pancreatic insufficiency, and glucose intolerance and had the remnant main pancreatic duct due to PJS based on imaging findings.

### Endoscopes

A forward-viewing endoscope (GIF-H290, GIF-2TQ260M, PCF-PQ260I, PCF-H290I, PCF-H290ZI, CF-HQ290ZI, TGF-UC260J, and SIF-H290S; Olympus Medical Systems, and EN450BI5, and EI530B; Fujifilm Medical, Tokyo, Japan) was used in all sessions.

### ERPD procedure

**Figure 2:**
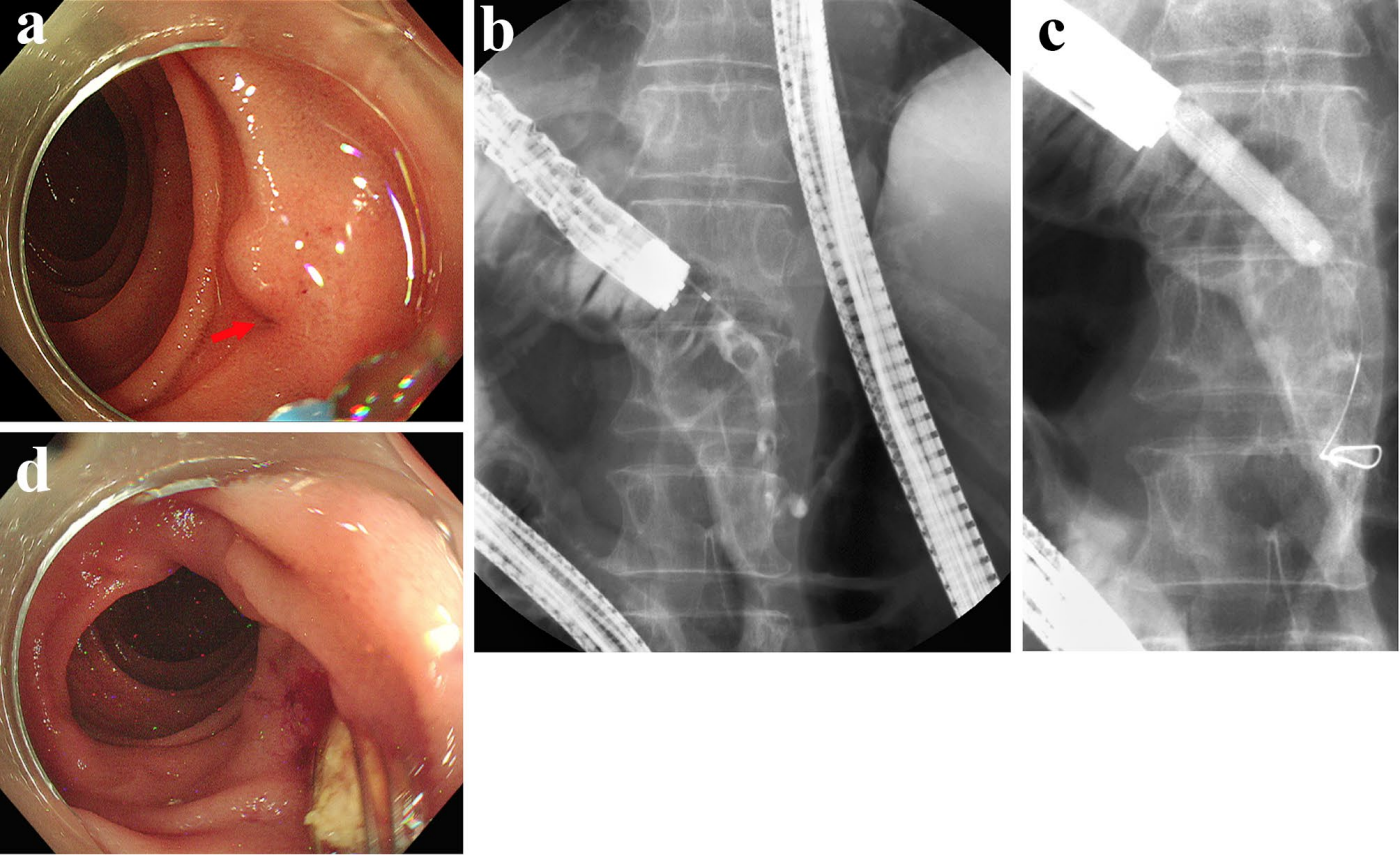
The procedures of endoscopic retrograde pancreatic drainage. (**a**) Endoscopy revealed a pinhole-like opening at the anastomotic site. There were no apparent tumor lesions. The red arrow shows the anastomotic site. (**b**) Two pancreatic calculi were identified in the remnant main pancreatic duct on endoscopic retrograde pancreatography. (**c**) Balloon dilation was performed at the anastomotic site to achieve waist disappearance. (**d**) Pancreatic calculi were removed using baskets.

When the anastomotic site was able to be identified, ERP was performed using a contrast cannula (TandemXL, and Swish ERCP cannula; Boston Scientific Japan, Tokyo, Japan, and PR-109Q, PR-110Q, and PR-V234Q; Olympus Medical Systems, and MTW ERCP-Catheter; MTW Endoscopie Manufaktur, Wesel, Germany). After ERP, a guidewire (VisiGlide, and VisiGlide2 angle; Olympus Medical Systems, and Roadrunner; Cook Medical, Wilston-Salem, NC, United States, and M-Through; Medico’s Hirata, Tokyo, Japan) was placed in the pancreatic duct. When ERP was unable to be directly applied, WGT was concomitantly employed. When cannulation was challenging due to obstruction of the anastomotic site, making an incision in the anastomotic site using a needle knife was effective. After placing the guidewire in the pancreatic duct, the anastomotic site was dilated using a contrast cannula or balloon dilator (MaxPass; Olympus Medical Systems, and Hurricane RX Biliary Balloon Dilatation Catheter, and CRE PRO GI Wireguided; Boston Scientific Japan, and ZARA EPBD balloon; KANEKA, Tokyo, Japan) or a dilator (ES dilator; Zeon Medical, Tokyo, Japan). When pancreatic calculi were present, they were removed using a basket catheter (Xemex lithotripsy basket; Zeon Medical, and Memory II 8 Fr Eight Wire Double Lumen Baskets; Cook Medical) or electrohydraulic lithotripsy by pancreatoscopy (SpyGlass DS; Boston Scientific Japan). Lastly, a pancreatic stent with or without an internal-flap (Geenen Pancreatic Stent, and Zimmon Pancreatic Stent; Cook Medical, and Advanix Pancreatic Stent; Boston Scientific Japan) was placed. The procedure of ERPD for a case of sPJS with pancreatic calculi is shown in Fig. 2. ERPD procedures were performed by 6 specialists certified by the Japan Gastroenterological Endoscopy Society in our group.

### Evaluation of clinical data and definitions of outcome variables

Clinical data were obtained from medical records of patients. The following characteristics of patients were evaluated: age, sex, primary disease for which the surgery was performed, surgical procedure and reconstruction method employed, causes of PJS, and symptoms of PJS.

Technical success of ERPD was considered if (1) The endoscope reached the anastomotic site and the anastomotic site was identified, (2) the main pancreatic duct was confirmed on pancreatography and a guidewire was placed, and (3) the anastomotic site was dilated and a pancreatic stent was placed as necessary.

The degree of dilation was evaluated based on the ratio of the maximum diameter of the balloon with respect to the maximum diameter of the remnant main pancreatic duct proximal to the anastomotic site before balloon dilation. Waist disappearance was also considered in the evaluation of balloon dilation. The remaining waist was confirmed by fluoroscopy. Lastly, the type (no-internal-flap or with internal-flap) and size of the pancreatic stents were evaluated.

Clinical success of ERPD was defined as having no recurrent sPJS during the follow-up period after ERPD. Recurrent sPJS was defined as having imaging findings of dilation of the remnant main pancreatic duct and clinical symptoms during the follow-up period, which required an endoscopic re-intervention.

Lastly, the following AE associated with ERPD were examined: bleeding and perforation due to scope insertion, and bleeding, perforation, and PEP due to ERPD. The severity of these AE was graded according to the American Society for Gastrointestinal Endoscopy guidelines^21^.

### Statistical analysis

The recurrence-free time after ERPD was calculated from the day of first ERPD to the date of first recurrence or the last day of the follow-up period. Cumulative recurrence was calculated using the Kaplan–Meier method and differences in the risk factors of sPJS recurrence were evaluated using the log-rank test. The following factors were examined as risk factors for sPJS recurrence: age, sex, characteristics of the anastomotic site, pancreatic stent placement, type and size of pancreatic stent, ERPD-related AE, and pancreatic stent migration. A *p*-value of < 0.05 was considered to be significant and all tests were two-sided. All statistical analyses were carried out using the SPSS statistical software program package (SPSS version 26.0 for Windows).

### Ethical statements

This study was approved by the ethics committees of Toyama Prefectural Central Hospital (No. 58-115) and Saiseikai Kanazawa Hospital (No. R2-9), and was conducted in accordance with the ethical standards described in the latest revision of the Declaration of Helsinki.

### Patient consent for patient participation and publication

Informed consent for patient participation and publication was received in the form of an opt-out in-hospital notice.

## Data Availability

All data relevant to the study are included in the article.

## Abbreviations

PJS: Pancreaticojejunal anastomotic stenosis
PD: Pancreaticoduodenectomy
sPJS: Symptomatic pancreaticojejunal anastomotic stenosis
ERPD: Endoscopic retrograde pancreatic drainage
EUS-PD: Endoscopic ultrasound-guided pancreatic duct drainage
AE: Adverse events
PpPD: Pylorus-preserving pancreaticoduodenectomy
SSpPD: Subtotal stomach-preserving pancreaticoduodenectomy
ERP: Endoscopic retrograde pancreatography
WGT: Wire-guided technique
PEP: Post endoscopic retrograde pancreatography pancreatitis
